# Bioaccessibility and Antioxidant Capacity of Grape Seed and Grape Skin Phenolic Compounds After Simulated In Vitro Gastrointestinal Digestion

**DOI:** 10.1007/s11130-024-01164-z

**Published:** 2024-03-19

**Authors:** Edurne Elejalde, María Carmen Villarán, Argitxu Esquivel, Rosa María Alonso

**Affiliations:** 1 TECNALIA, Basque Research and Technology Alliance (BRTA), Parque Tecnológico de Álava, C/Leonardo Da Vinci 11, 01510 Miñano, Álava Spain; 2 FARMARTEM Group. Analytical Chemistry Department, Faculty of Science and Technology, University of the Basque Country (UPV/EHU), Barrio de Sarriena, S/N, 48940 Leioa, Bizkaia Spain

**Keywords:** Grape skin, Grape seed, Polyphenols, Bioaccessibility, Antioxidant capacity, In vitro gastrointestinal digestion

## Abstract

Grapes present recognized beneficial effects on human health due to their polyphenolic composition. The grape overproduction together with the wine sales down and the world socioeconomic situation makes the wine grape valorization a promising strategy to give an added-value to this natural product. The objective of the present work was to study the influence of in vitro gastrointestinal digestion on antioxidant capacity and polyphenolic profile of skin and seed extracts of different grape varieties (Tempranillo, Graciano, Maturana tinta and Hondarrabi zuri). After in vitro gastrointestinal digestion, total phenolic content (TPC) of seed polyphenolic extracts decreased significantly for all the varieties. The highest decrease was for Tempranillo going from 108 ± 9 to 50 ± 3 mg / g dry matter (dm). This variety also showed the highest decrease of 90% in antioxidant capacity. However, for all the skin polyphenolic extracts there was an increase in TPC. The highest variation was also for Tempranillo. It varied from 10.1 ± 0.8 to 55.1 ± 0.9 mg / g dm. Among red varieties Tempranillo skin polyphenolic extract showed the lowest undigested anthocyanin content but the highest bioaccessibility index (BI) of 77%. For flavanols, flavonols and procyanidins the seed polyphenolic extracts showed a BI at the intestinal phase between 11% for (+)-epicatechin gallate to 130% procyanidin A2. The results of this study suggest that grape skin extracts and grape seed extracts are a reliable source of bioaccessible antioxidant polyphenols, to be used for the development of antioxidant supplements with specific functionalities depending on the grape variety.

**Supplementary Information:**

The online version contains supplementary material available at 10.1007/s11130-024-01164-z.

## Introduction

Polyphenols constitute a group of phytochemicals widely distributed in the plant kingdom with beneficial effects in the prevention of many noncommunicable diseases, such as cardiovascular diseases, diabetes and cancer [1–3]. Polyphenols are strong antioxidants and act as a defense against oxidative stress caused by excess reactive oxygen species. Some of the antioxidant, anti-inflammatory and anticarcinogenic capacity of the polyphenols and their metabolites might also be attributed to their ability to modulate intestinal microbiota population, having a prebiotic-like effect. The prebiotic-like activity arises from their ability to produce variations in the microflora community, inhibiting detrimental bacteria, stimulating beneficial bacteria and maintaining a healthy and resilient microbiota [4].

Commonly, grapes are recognized as natural food with a high content of polyphenolic compounds [5] and consequently, a very interesting source for the development of food supplements. The worldwide grape overproduction due to the dry and hot meteorological conditions for the last years, the decrease of global wine consumption accentuated by the COVID-19 pandemic and the associated energy crisis, makes the wine grape valorization a promising strategy to give an added-value to this healthy natural food [6]. In fact, antioxidant supplements containing grape polyphenols have received much attention by elite and recreational athletes for example, as a strategy to reduce oxidative stress induced by intense exercise and to promote muscle recovery [7]. The amount of polyphenols in grapes is affected, but not limited, by variety, genotype, cultivation practices, vineyard characteristics (soil, sanitary stage) and climatic factors [8, 9]. Besides, grape polyphenols are heterogeneously distributed in the stalk, skin, pulp and seeds. Despite the evident bioactivity of polyphenols, it is relevant to first consider their bioaccessibility [10]. It refers to polyphenols released from the matrix by the action of the gastrointestinal enzymes, whereas bioavailability considers the absorbed compounds from the gastrointestinal tract and their transportation to body tissues [11].

In vitro methods simulating digestion processes are widely used to study the gastrointestinal effect on food components [12]. Although human nutritional studies are considered “gold standard”, simulated in vitro digestion methods have the advantage of being more rapid, less expensive, less labour-intensive and do not have ethical restrictions [13]. In this study the standarised static in vitro digestion was conducted following the guidelines of the widely accepted methodology developed during the COST action INFOGEST [14] The aim of this study was to investigate the effect of in vitro gastrointestinal digestion on the polyphenolic composition and antioxidant capacity of skin and seeds of different grape varieties to obtain a reliable estimation of their potential for the development of antioxidant food supplements. Considering the importance of the grape sector worldwide, the knowledge of these data is mandatory to collaborate in offering an added value to this natural product.

## Materials and Methods

This section is described in the Supplementary Information.

## Results and Discussion

### Effect of In Vitro Gastrointestinal Digestion of Grape Skins and Grape Seeds on Total Phenolic Content and Antioxidant Capacity

**Fig. 1:**
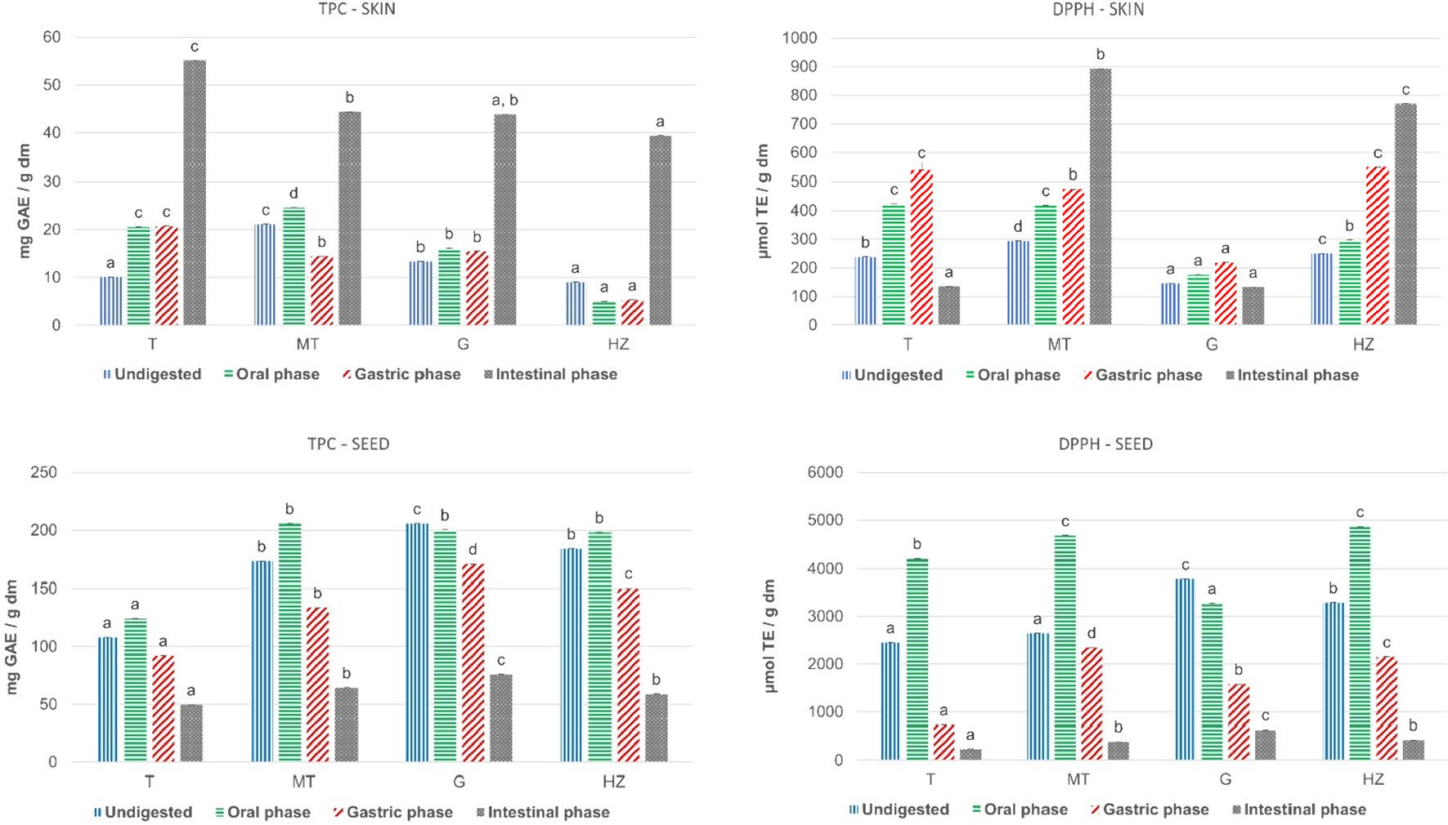
Changes in total phenolic content (TPC) and antioxidant capacity (DPPH) during in vitro gastrointestinal digestion of different skin and seed grape varieties. *Results are expressed as mean ± standard deviation (*n* = 3). Different letters in the same stage of the in vitro gastrointestinal digestion represent significant difference among the grape varieties (*p* < 0.05). TPC, expressed as gallic acid equivalents (GAE) mg *per* g dry matter (dm). DPPH, expressed as μmol trolox equivalents (TE) *per* g dry matter (dm). Dry matter (dm) is referred to the polyphenolic extract

The results reported in Fig. 1 show the effect of every stage of in vitro gastrointestinal digestion on TPC and antioxidant capacity (DPPH) of polyphenolic skin and seed extracts of different grape varieties commonly grown in the north of Spain. These are three red grape varieties: Tempranillo (T), Maturana tinta (MT) and Graciano (G), and one white grape variety: Hondarrabi zuri (HZ).

The mean TPC of undigested seed extracts was 12.5 times higher than the TPC of undigested skin extracts. The TPC value of undigested skin extracts varied in the interval 9.1 – 21.1 mg GAE / g dm and for seed extracts in the interval 108 – 206 mg GAE / g dm. These results in TPC content in skin and seed extracts are compatible with previous studies [15] with TPC values between 33.5 to 37.5 mg GAE / g dm for skin extracts (red grape varieties) and 73.7 to 107.8 mg GAE / g dm for seed extracts (red grape varieties). Other authors [16] showed for 11 red grape seed extracts a TPC interval of 79.2 – 154.6 mg GAE / g dm. MT was the skin extract with the highest TPC, while for the seed extracts G was the variety with the highest value. After in vitro gastrointestinal digestion there was a significant increase in TPC for the skin extracts, independently the grape variety. It varied in the interval 210% for MT skin extract to 547% for T skin extract. However, for seed extracts and independently the grape variety, the TPC value decreased significantly in the interval 32% for HZ extract and 46% for T extract. Other authors have also reported this increase for skin extracts and decrease for seed extracts [17].

Regarding DPPH, the mean antioxidant capacity of the undigested seed extracts was 13.1 times higher than the antioxidant capacity of skin extracts. This difference is in concordance with TPC results, which results with a direct correlation between TPC and DPPH suggesting that the total polyphenolic content influences significantly the antioxidant capacity of grape seed extracts. Guendez et al. [18] showed also a significant correlation between DPPH scavenging activity and the total polyphenol content of several grape seed extracts from different varieties. After in vitro gastrointestinal digestion, and for all the seed extracts the radical scavenging activity measured by DPPH method decreased from undigested samples to intestinal phase, with final values of antioxidant capacity much lower than those for undigested samples. The lowest decrease was of 83% for G and of 90% for T, showing slight differences among seed varieties. These results are compatible with the TPC decrease from the undigested samples during in vitro gastrointestinal digestion. The interval of this reduction was 54% for T grape seed extract to 68% for that of HZ. Since the DPPH reduction is much higher than the TPC decrease, these results show that the polyphenols are highly sensitive to alkaline conditions such as in the intestinal phase, hence, a proportion of these compounds might be metabolized to give rise to compounds with different chemical and antioxidant properties. However, for skin extracts the evolution of DPPH after the in vitro gastrointestinal digestion was heterogeneous. In this regard, for G skin extract there was a light reduction of 9% and for T skin extract, the reduction of DPPH was of 44%. These results are opposite to those of the other two grape varieties with a DPPH increase of the skin extract after the in vitro gastrointestinal digestion of 204% for MT and of 208% for HZ grape varieties. It was a surprising result that T and G skin extracts showed a reduction in antioxidant capacity while the TPC value was increased in these samples, which is in accordance with the results of other studies [17]. The decrease in the radical scavenging capacity may arise from the specific modification in the polyphenolic composition in this grape varieties compared to undigested samples. pH variations may also cause a modification in the antioxidant capacity and the interference of antioxidant polyphenols with other components of the extracts may also result in differences in the bioaccessibility.

### Changes in Polyphenolic Composition in Grape Skins and Grape Seeds During In Vitro Gastrointestinal Digestion

In order to evaluate the bioaccessibility of individual phenolic compounds in grape polyphenolic extracts during in vitro gastrointestinal digestion, eleven compounds were selected based on their tested benefits for sport performance and their relevant presence in grape skin and seeds. Concerning anthocyanins, present almost exclusively in red grape variety skins, three of the main monoglucoside anthocyanidins were analyzed: malvidin-3-*O*-glucoside (malv-3-gluc), delphinidin-3-*O*-glucoside (delp-3-gluc) and cyanidin-3-*O*-glucoside (cyan-3-gluc). Several studies suggest that consuming anthocyanin-rich foods leads to faster recovery of markers of exercise-induced muscle damage after resistance training and endurance events [19, 20]. Flavanols are the most abundant phenolic family in grape seeds [21] and many investigations have revealed their benefits on sport performance [22, 23]. In this case, the mayor grape flavanols: (+)-catechin, (−)-epicatechin, epicatechin gallate were quantified. Other important grape seed polyphenols are the proanthocyanins, oligomers in galloylated and no-galloylated form of flavanols [24]. Besides, recovery studies after eccentric exercise using supplements have shown that flavonols like quercetin, exert positive effects on sports performance [25]. In this research, two of the principal flavonols present in grape seeds were analyzed: quercetin-3-glucoside and quercetin-3-rutinoside, known as rutin. Regarding procyanidins, they derive from proanthocyanidins (condensed tannins). In *Vitis vinifera* grapes procyanidins are mainly oligomers and polymers of (+)-catechin and (−)-epicatechin linked through C4\C8 bonds [8]. Procyanidins can be categorized into A-type and B-type depending on the stereo configuration and linkage between monomers. B-type are the most abundant, with B1 and B2 occurring most frequently. Related with A-type procyanidins the most common are A1 and A2 [26]. In this study, procyanidin B1, B2 and A2 were analyzed.

The analysis of all compounds was carried out using ultra-performance liquid chromatography-tandem mass spectrometry (UPLC-MS/MS).

**Fig. 2:**
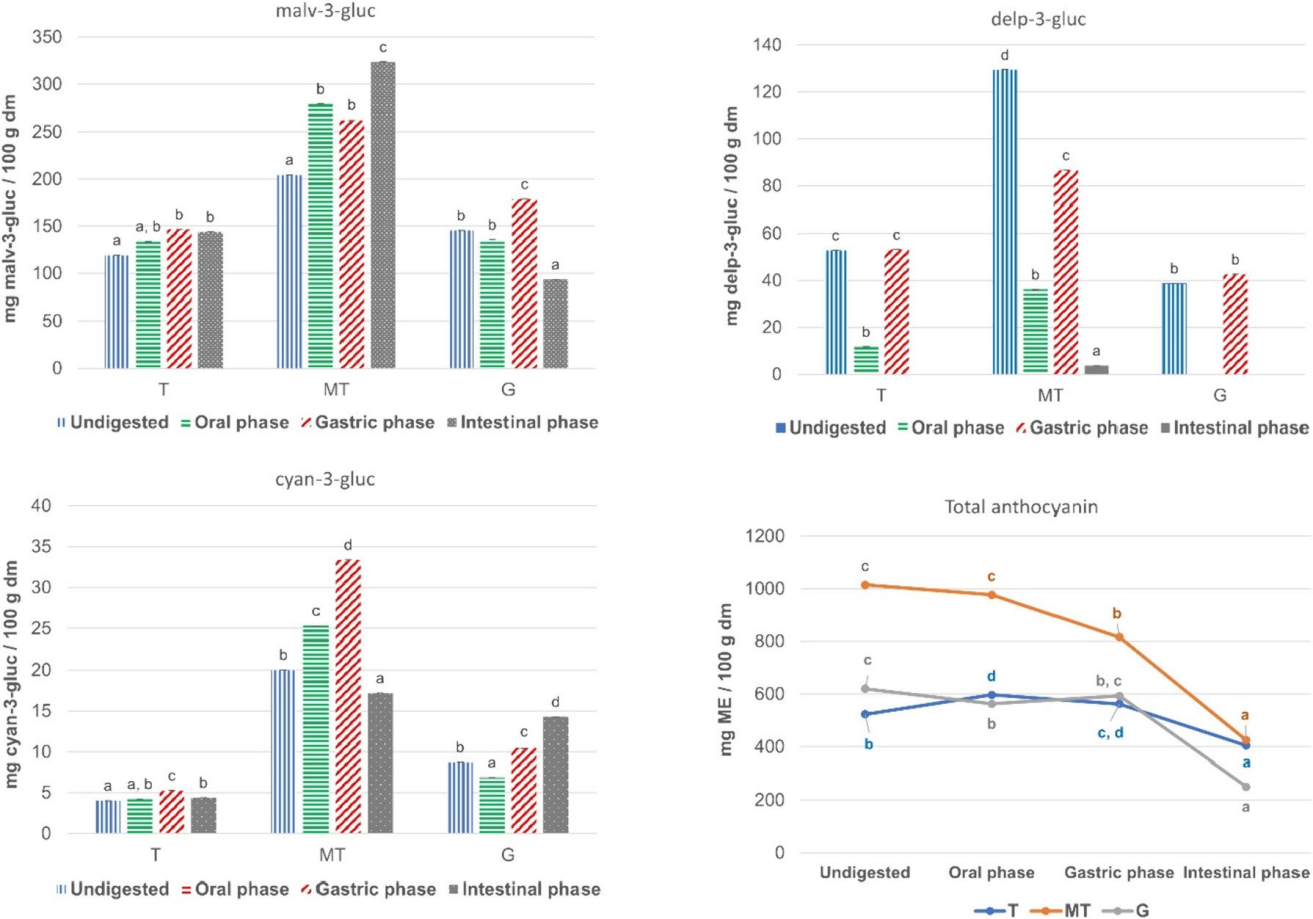
Concentrations (mg/100 mg dm) of malv-3-gluc, delp-3-gluc, cyan-3-gluc and total anthocyanins during in vitro gastrointestinal digestion in skin polyphenolic extracts of T, MT and G grape varieties. *Results are expressed as mean ± standard deviation (*n* = 3). Different letters in the same stage of the in vitro gastrointestinal digestion represent significant difference among the grape varieties (*p* < 0.05). Total anthocyanin was expressed as malvidin–3-*O*-glucoside equivalents (ME) mg *per* g dry matter (dm). Dry matter (dm) is referred to the polyphenolic extract

Considering total anthocyanins (TA), although the highest anthocyanin content was found for MT skins (see Fig. 2), this variety showed low bioaccesibility index (BI) after the in vitro gastrointestinal digestion as shown in Table 1.

**Table 1:** Bioaccessibility index of anthocyanins in skin polyphenolic extracts of different grape varieties during in vitro gastrointestinal digestion

		T	MT	G
Anthocyanin	Gastrointestinal phase	BI	BI	BI
Malvidin-3-*O*-glucoside	Oral	112%	136%	93%
	Gastric	123%	128%	123%
	Intestinal	121%	158%	64%
Delphinidin-3-*O*-glucoside	Oral	22%	28%	
	Gastric	101%	67%	110%
	Intestinal		3%	
Cyanidin-3-*O*-glucoside	Oral	104%	127%	78%
	Gastric	131%	167%	120%
	Intestinal	109%	86%	163%
Total anthocyanins	Oral	114%	96%	91%
	Gastric	107%	80%	96%
	Intestinal	77%	42%	40%

Bioaccessibility index (BI) is calculated as percentage of the concentration of the compound after every in vitro gastrointestinal phase respect to and the concentration of the compound in the undigested sample

Regarding T skin, with the lowest initial TA content, resulted with the highest BI at the intestinal phase. G skin showed a similar evolution to MT during the in vitro gastrointestinal digestion. Several authors [27] have proposed that this reduction could be attributed to the cleavage of the sugar moiety producing the aglycones that are not detected by our chromatographic method. Regarding MT and T skin extracts there was a significant increase of malv-3-gluc at the end of the in vitro gastrointestinal digestion. For G extracts however, the intestinal stage showed a reduction in malv-3-gluc. In relation to cyan-3-gluc, G showed a significant increase during the in vitro gastrointestinal digestion; however, a significant decrease after the intestinal digestion was observed for MT skin extracts. For delp-3-gluc the evolution was similar for all the grape skin extracts showing a significant decrease in the oral phase, the highest bioaccessibility in the gastric phase and a notable reduction in the intestinal phase.

Kamonpatana et al. [28] suggested that the highest hydroxylation on the B-ring could result in a decrease on the structural stability delp-3-gluc<cyan-3-gluc<malv-3-gluc. However, the gastric phase causes an increase in some anthocyanins only in T and MT varieties. This effect might be explained by the pH drop due to the gastric digestion since anthocyanins exist basically in the flavylium form at pH below 2 and as pH increases at the intestinal stage there is a deprotonation producing quinoidal forms that cannot be detected under our chromatographic conditions. These results show that the in vitro gastrointestinal digestion influences the anthocyanin bioaccessibility and therefore, their bioavailability. The selection of the grape variety is consequently essential to consider the skin as an anthocyanin source for the development of antioxidant supplements based on grape polyphenols.

For seed extracts, the concentrations of three flavanols (catechin, epicatechin and epicatechin gallate), two flavonols (quercetin-3-glucoside and quercetin-3-rutinoside) and three procyanidins (B1, B2 and A2) during the in vitro gastrointestinal digestion are collected in Table 2.

**Table 2:** Concentrations of phenolic compounds in grape seed extracts of different grape varieties during in vitro gastrointestinal digestion

Phenolic compound	Gastrointestinal phase	Tempranillo			Maturana tinta			Graciano			Hondarrabi zuri		
		Mean	SD	BI	Mean	SD	BI	Mean	SD	BI	Mean	SD	BI
(+)-Catechin	Undigested	15.8	0.7^b^		81	3^c^		214	11^b^		374	17^d^	
	Oral	14.5	0.8^b^	91%	40	2^a^	50%	116	7^a^	55%	103	6^a^	28%
	Gastric	21	3^c^	134%	62	8^b^	77%	196	26^b^	92%	316	44^c^	85%
	Intestinal	10.6	0.5^a^	67%	37	2^a^	46%	112	5^a^	53%	158	7^b^	42%
(−)-Epicatechin	Undigested	18.4	0.5^a,b^		66	2^c^		421	10^b^		600	14^c^	
	Oral	21.4	0.1^b^	117%	37.9	0.3^a^	57%	280	2^a^	67%	249	2^a^	42%
	Gastric	26	4^c^	142%	57	8^b^	86%	423	57^b^	101%	492	67^b^	82%
	Intestinal	15.0	0.1^a^	82%	32.5	0.4^a^	49%	236.4	0.8^a^	56%	262	2^a^	44%
(−)-Epìcatechin gallate	Undigested	12.5	0.6^b^		32	1^d^		42	2^c^		126	6^d^	
	Oral	12.5	0.4^b^	101%	18.5	0.3^b^	58%	26.7	0.7^b^	63%	30	1^b^	24%
	Gastric	15	1^c^	123%	27	2^c^	85%	40	3^c^	94%	72	7^c^	57%
	Intestinal	3.4	0.3^a^	27%	6.1	0.7^a^	19%	7.7	0.6^a^	18%	13	2^a^	11%
Quercetin-3-glucoside	Undigested	1.4	0.1^a^		1.4	0.1^a^		2.1	0.1^b^		1.9	0.1^c^	
	Oral	2.6	0.3^b^	186%	1.9	0.3^b^	138%	2.3	0.2^b^	113%	1.3	0.2^b^	68%
	Gastric	3.7	0.2^c^	268%	3.4	0.3^c^	238%	3.8	0.3^c^	183%	1.5	0.1^b^	77%
	Intestinal	1.7	0.1^a^	122%	1.4	0.2^a^	99%	1.6	0.2^a^	80%	0.8	0.1^a^	41%
Quercetin-3-rutinoside	Undigested	0.7	0.1^a^		nd			nd			nd		
	Oral	1.3	0.1^b^	183%	nd			nd			nd		
	Gastric	2.3	0.3^c^	329%	nd			nd			nd		
	Intestinal	0.9	0.1^a^	129%	nd			nd			nd		
Procyanidin B1	Undigested	61.1	0.3^c^		225	1^d^		242	1^d^		229.3	0.7^c^	
	Oral	45.1	2.1^b^	74%	137	6^b^	61%	136	6^b^	56%	203	10^b^	89%
	Gastric	65	6^c^	107%	187	13^c^	83%	182	16^c^	75%	260	22^d^	113%
	Intestinal	21.8	0.2^a^	36%	98.1	0.6^a^	44%	99	1^a^	41%	142.3	0.4^a^	62%
Procyanidin B2	Undigested	74.4	0.2^b^		163.7	0.3^d^		475	2^d^		443.8	0.5^d^	
	Oral	71.3	0.1^b^	96%	118.2	0.2^b^	72%	301	1^b^	63%	277.7	0.3^b^	63%
	Gastric	80	7^c^	108%	136	12^c^	83%	339	29^c^	71%	308	28^c^	69%
	Intestinal	27.8	1.4^a^	37%	73	4^a^	45%	213	11^a^	45%	180	10^a^	41%
Procyanidin A2	Undigested	4.2	0.1^a^		4.8	0.1^a^		4.6	0.1^a^		3.9	0.1^a^	
	Oral	6.9	0.4^b^	165%	6.3	0.1^a^	132%	6.1	0.3^a^	131%	nd		
	Gastric	10	_1_c	257%	nd			11	_1_b	236%	nd		
	Intestinal	3.9	0.5^a^	94%	nd			6.1	0.7^a^	130%	nd		

Results are expressed as mean ± standard deviation (*n* = 3) in mg / 100 g dm. Different letters (a, b, c, d) for each compound in the same column and grape variety represent significant difference among in vitro gastrointestinal digestion phases. Bioaccessibility index (BI) is calculated as percentage of the concentration of the compound after every in vitro gastrointestinal phase respect to the concentration of the compound in the undigested sample. Dry matter (dm) is referred to the polyphenolic extract

All the identified compounds in the seed extracts showed a BI between 11 – 130% after the in vitro gastrointestinal digestion. For the flavanols, the grape seed variety with the highest flavanol content was the white variety HZ, followed by the red varieties G, MT and T. Epicatechin was the major flavanol with an initial concentration of 600 ± 14 mg / 100 g dm for HZ to 18.4 ± 0.5 mg / 100 d dm for T. For all the varieties, concentrations of flavanols were higher in the gastric phase than in other phases of digestion. Some authors [29] have attributed these differences to the relatively poorer stability of flavanols under neutral or near-alkaline conditions than under acidic environment. T variety showed the highest BI, indicating that the grape variety rather than flavanol content among other factors exerts influence on bioaccessibility. These results are in accordance with other authors [17, 30] who also showed a higher concentration for flavanols in seeds of white grape varieties, but a higher bioaccessibility of these compounds after in vitro gastrointestinal digestion for the red grape varieties.

In general, the concentration of the individual flavonol glycosides detected was not affected after the in vitro gastrointestinal digestion and showed a good stability at the in vitro gastrointestinal conditions, as reported by other authors [31]. Quercetin-3-glucoside was the flavonol detected in all the seed grape varieties. Some studies [32] have reported that quercetin glucosides are more efficiently absorbed than quercetin itself. The G red seed variety extract with the HZ white seed variety extract showed the highest concentration followed by T and MT. Regarding rutin, only in T extract was detected, showing a high stability after in vitro gastrointestinal digestion as same as quercetin, as also described by Gayoso et al. [33].

Regarding procyanidins for the undigested samples MT showed the highest concentration for B1, while in T, G and HZ procyanidin B2 was predominant. G showed the highest concentration. Procyanidin A2 showed a lower concentration for all the grape seed varieties. These results are in accordance with other authors [34] who reported 4.7 ± 1.8 mg / g Merlot noir grape variety seed and 5.7 ± 2.1 mg/ g Cabernet sauvignon grape seed. After in vitro gastrointestinal digestion the procyanidin B1 and B2 level was significantly decreased (*p* < 0.05) for all the varieties. For B1 this decrease interval was from 64% for T to 38% for the white seed variety HZ. It must be pointed out that after the in vitro gastrointestinal digestion the BI resulted very similar for all the red grape seed extracts (36, 44 and 41% for T, MT and G, respectively) independently the initial undigested concentration. For B2, the BI after the in vitro gastrointestinal digestion was similar for all the seed extracts and varied from 37% for T to 45% for MT and G, in accordance with other authors [35] who also reported a decrease of B1 and B2 after intestinal digestion.

## Conclusions

The results highlight that the bioaccessibility of grape skin and seed polyphenols depends on the grape variety. The in vitro gastrointestinal digestion conditions with a relevant influence of pH and the applied enzymes affects the stability and the bioaccessibility of the polyphenols producing variations in the concentration and the polyphenolic profile. Despite the significant loss of the different polyphenols from the skin or the seed during the digestion the extracts still have antioxidant capacity. Concentrations of anthocyanins, flavanols, flavonols and procyanidins and the antioxidant capacity in digested samples were maintained suggesting high bioaccessibility of most phenolic compounds. The characteristics of the grape skin and seeds of each variety pointed out specific bioaccessibility of the different polyphenols that may be considered for the development of antioxidant supplements. Efforts should be focused now on studying the bioavailability of the bioaccessible polyphenols obtained from grape skin and grape seed extracts in cell models to study the absorption efficiency into blood. Understanding the antioxidant supplements’ role requires a deeper knowledge of their route through the digestive system.

### Supplementary Information

Below is the link to the electronic supplementary material.

Supplementary file1 (DOCX 50 KB)

## Data Availability

No datasets were generated or analysed during the current study.
