# Supplementary material for: Bioaccessibility and Antioxidant Capacity of Grape Seed and Grape Skin Phenolic Compounds After Simulated In Vitro Gastrointestinal Digestion
Source: Plant Foods Hum Nutr. 2024 Mar 19;79(2):432–9. doi: 10.1007/s11130-024-01164-z (PMC11178585; doi:10.1007/s11130-024-01164-z)
Supplement: Supplementary file 1 — Supplementary file1 (DOCX 50 KB) [file 11130_2024_1164_MOESM1_ESM.docx]

**Supplementary Information (SI)**

**Bioaccessibility and Antioxidant Capacity of Grape Seed and Grape Skin Phenolic Compounds after Simulated *In vitro* Gastrointestinal Digestion**

(Plant Foods for Human Nutrition)

Edurne Elejalde^1^ <https://orcid.org/my-orcid?orcid=0000-0002-8125-4445> • María Carmen Villarán^1^ <https://orcid.org/0000-0002-0074-4224> • Argitxu Esquivel^1^<https://orcid.org/0000-0001-9091-4532> • Rosa María Alonso^2^ <https://orcid.org/0000-0001-9046-6602>

^1^ TECNALIA, Basque Research and Technology Alliance (BRTA)**,** Parque Tecnológico de Álava, c/Leonardo Da Vinci 11, 01510 Miñano (Álava), Spain

^2^ FARMARTEM group. Analytical Chemistry Department, Faculty of Science and Technology, University of the Basque Country (UPV/EHU), Barrio de Sarriena, s/n, 48940 Leioa (Bizkaia), Spain

***** Corresponding author: Edurne Elejalde [edurne.elejalde@tecnalia.com](mailto:edurne.elejalde@tecnalia.com)

**Materials and Methods**

**Samples**

Three native red and one native white grape varieties (*Vitis vinifera*) were selected. The grapes were initially destined for winemaking. The grape bunches were directly picked in the vineyard during 2021 season at their optimal quality and maturity point and taken to the laboratory to be immediately frozen in liquid nitrogen. The grapes were manually separated into skin and seeds which were afterwards freeze dried (Lyobeta 25; Telstar, Terrassa, Barcelona, Spain) until extraction.

**Preparation of the polyphenolic extracts**

The conditions for the ultrasound-assisted extraction were established according to the literature [1, 2]. Extraction was performed using 10 g of powdered sample (skin or seed) with 500 mL of ethanol at 50 % v/v in an ultrasound beaker with a sonde standard of 25 mm (Bioblock Scientific Vibra Cell VCX 750, Fisher Scientific, Madrid, Spain) and sonicated for 20 minutes. After the extraction, the polyphenolic extract was centrifuged (5810R Eppendorf; Merck Life Science, Madrid, Spain) for 5 minutes at 4000 rpm and filtrated through 0.45 µm filter. Ethanol was eliminated by a rotatory evaporator and the aqueous extract was lyophilized and stored until analysis.

***In vitro* simulated digestion**

Standardized static *in vitro* simulated digestion to the polyphenolic extracts of grape skins and seeds was conducted following the guidelines of INFOGEST methodology which includes oral, gastric and intestinal phases [3]. After each phase was completed, a sample was taken and preserved at -80 ºC. for further analyses. The simulated digestions were done by triplicate.

**Bioaccessibility index**

The percentage of bioaccessible polyphenolic compounds during the gastrointestinal digestion was calculated using the bioaccessibility index [4], according to the following equation:

$$\mathrm{Bioaccessibility}index (\%)= (CC digested/CC undigested) x 100$$

Where, CC digested is the concentration of the compound/compounds after the *in vitro* oral, gastric or intestinal digestion phase and CC undigested is the concentration of the compound/compounds before the *in vitro* gastrointestinal digestion.

**Determination of Total Phenolic Content (TPC)**

The TPC was determined using Folin-Ciocalteu assay [5]. 20 µL of appropriately diluted sample (grape skins polyphenolic extract or grape seeds polyphenolic extract) was mixed thoroughly with 100 µL of 10 % Folin-Ciocalteu’s phenol reagent in wells of a 96-well microplate. 80 µL sodium carbonate solution of 75 g/L were added. After 90 min in darkness at room temperature, the absorbance was measured at 750 nm in the microplate photometer (Multiskan™ FC; Fisher Scientific, Madrid, Spain). The concentration was expressed as milligrams of gallic acid equivalents (GAE) per g of dry matter (dm) polyphenolic extract based on a standard curve of gallic acid.

**Total Anthocyanin analysis (TA)**

The TA method was applied according to Di Stefano et al. [6]. For the analysis, the sample (grape skins polyphenolic extract) was diluted with a solution of ethanol:water:hydrochloric acid (70:30:1, v/v/v) and the absorbance was measured immediately at 540 nm in a spectrophotometer (Lambda 365 UV/VIS; Perkin Elmer, Madrid, Spain). The final result was expressed as malvidin-3-*O*-glucoside equivalents (ME) in mg per 100 grams of dry matter (dm) polyphenolic extract calculated from the following equation: C (mg/L) = A540 nm * 26.6 *d where, A540 nm is the absorbance at 540 nm and d is the applied dilution.

**Antioxidant capacity determination-DPPH radical scavenging activity**

The scavenging capacity of the polyphenolic extract (from skins or seeds) was evaluated by DPPH method based on the stability of 2, 2-diphenyl-1-picrylhydrazyl radical with some modifications [7]. 50 µL of appropriately diluted sample were added to 2.950 mL of 0.1 mM methanolic (80 %) DPPH radical solution, vortex mixed and incubated in dark for 30 min at room temperature. After incubation, the absorbance was measured at 515 nm in a spectrophotometer. Trolox was used as the reference compound. The results are expressed in mmol or µmol trolox equivalents (TE) per 100 g dry matter (dm) polyphenolic extract.

**UPLC-MS analysis of polyphenolic profiles**

UPLC-MS/MS technology was used to quantify the most representative polyphenols in the samples (grape skins or grape seeds polyphenolic extracts). The analysis was performed on a Waters Acquity UPLC system coupled to a triple quadrupole mass spectrometer (TQD) equipped with an orthogonal Z-spray-electrospray ionization source (ESI) (all from Waters Corporation, Milford, MA, USA). The column was an Acquity UPLC BEH C18 (2.1 x 50 mm, 1.7 µm particle size from Waters).

For the analysis of anthocyanins in grape skins polyphenolic extracts (malvidin-3-*O*-glucoside, delphinidin-3-*O*-glucoside, cyanidin-3-*O*-glucoside), the composition of mobile phases was water 0.1 % formic acid (A) and, acetonitrile (B). The percentage of aqueous solvent A was changed as follow: 0 min, 95 % A; 5 min, 0 % A; 5.2 min, 95 % A; and, 7 min, 95 % A. The flow rate was 0.4 mL/min and the column temperature was 35 ºC. The injection volume was 2 µL.

For the analysis of flavanols ((+)-catechin, (-)-epicatechin and (-)-epicatechin gallate), flavonols (quercetin-3-glucoside and quercetin-3-rutinoside) and condensed tannins (procyanidin B1, B2, A2) in grape seed polyphenolic extracts, the mobile phases were water 0.1 % formic acid (A) and, acetonitrile 0.1 % formic acid (B). The elution gradient was: 0 min, 95 % A; 6.8 min, 75 % A; 7.9 min, 65 % A; 9.1 min; 5 % A; 10.2 min, 95 % A; and, 11.4 min, 95 % A. The flow rate of the mobile phase was 0.25 mL/min, the temperature of the column was 25 ºC and the injection volume was 2 µL.

Mass spectrometric conditions were optimized by infusing each compound. Drying gas as well as nebulizing gas was nitrogen and, collision gas was argon. For the analysis of anthocyanins, flavan-3-ols, and flavonols the mass spectrometry conditions were: desolvation gas flow: 800 L/h; source temperature: 130 ºC; desolvation temperature was 400 ºC, capillary voltage: 3.00 (ESI -) and 4.00 (ESI +) and the cone gas flow 60 L/h. A multiple reaction monitoring method (MRM) using retention windows was set up (see Table 1.).

The quantification for all the compounds was done with external calibration plots, constructed by linear regression from available phenolic standards.

**Statistical analysis**

Results in this study are reported as means ± standard deviation (SD) of at least three independent batches for each sample. Data were analyzed by one-way ANOVA and the Fisher‘s Least Significant Difference (LSD) test to estimate the differences between values for the sample tested, where statistical significance was declared at p < 0.05. Statgraphics Centurion XVII software was used for statistical analysis.

*References*

1. Figueiredo-González M, Martínez-Carballo E, Cancho-Grande B, et al (2012) Pattern recognition of three Vitis vinifera L. red grapes varieties based on anthocyanin and flavonol profiles, with correlations between their biosynthesis pathways. Food Chem 130:9–19. https://doi.org/10.1016/j.foodchem.2011.06.006

2. Natolino A, Da Porto C (2020) Kinetic models for conventional and ultrasound assistant extraction of polyphenols from defatted fresh and distilled grape marc and its main components skins and seeds. Chem Eng Res Des 156:1–12. https://doi.org/10.1016/j.cherd.2020.01.009

3. Brodkorb A, Egger L, Alminger M, et al (2019) INFOGEST static in vitro simulation of gastrointestinal food digestion. Nat Protoc 14:991–1014. https://doi.org/10.1038/s41596-018-0119-1

4. Ortega N, Maciá A, Romero M-P, et al (2011) Matrix composition effect on the digestibility of carob flour phenols by an in-vitro digestion model. Food Chem 124:65–71. https://doi.org/10.1016/j.foodchem.2010.05.105

5. Shahidi F, Zhong Y (2015) Measurement of antioxidant activity. J Funct Foods 18, Part B:757–781. https://doi.org/10.1016/j.jff.2015.01.047

6. Di Stefano R, Cravero MC, Gentilini N (1989) Metodi per lo studio dei polifenoli dei vini. Enotecnico 25:83–89

7. Brand-Williams W, Cuvelier ME, Berset C (1995) Use of a free radical method to evaluate antioxidant activity. LWT - Food Sci Technol 28:25–30. https://doi.org/10.1016/S0023-6438(95)80008-5
